# No Place for Poor Men: On the Asymmetric Effect of Urbanization on Life Satisfaction

**DOI:** 10.1007/s11205-022-02946-1

**Published:** 2022-05-28

**Authors:** Camilla Lenzi, Giovanni Perucca

**Affiliations:** Department of Architecture, Built Environment and Construction Engineering - Politecnico Di Milano, Piazza Leonardo da Vinci 32, 20133 Milano, Italy

**Keywords:** Urbanisation, Agglomeration, Borrowed size, Life satisfaction, R30, I31

## Abstract

The literature on the geography of subjective well-being largely converges in pointing out the occurrence, at least in developed countries, of an urban/rural divide: people living in the most urbanized regions tend to be significantly less satisfied than those living in rural areas. This paper aims at reassessing this statement by taking into consideration an important aspect, frequently overlooked in the literature, i.e. people-based characteristics. Individuals are not alike and may differently experience and appreciate the advantages and disadvantages of urbanisation. Characteristics such as the level of education, the type of occupation and, more generally, the income level can mediate the capacity to reap urbanisation advantages (as the accessibility to advanced services and diversified job markets) and mitigate urbanization disadvantages (such as cost of living and congestion). Additionally, but based on the same reasoning, more educated and affluent individuals (negatively) value distance from top rank centres more than less educated and affluent ones. We test and prove these propositions in a study on the subjective well-being of more than 250,000 individuals living in European cities, defined as NUTS3 regions, in the period 2004–2010.

**Supplementary Information:**

The online version contains supplementary material available at 10.1007/s11205-022-02946-1.

## Introduction

Within the long stream of research examining the determinants of individual subjective well-being initiated by the seminal paper of Easterlin 1974 (Dolan et al., 2008), an increasing literature is investigating the relationship between urbanization and subjective well-being.

The empirical findings, however, highlighted a puzzle. In fact, whereas cities give raise to the most intense processes of economic growth (Glaeser, 2011), urbanization is frequently associated with lower levels of well-being when compared with less dense settings (Graham, 2012). In short, there is a spatial mismatch between the objective and subjective dimensions of well-being, especially in most developed and affluent countries in the Western part of the globe (Burger et al., 2020).

The most diffused interpretation of the urban well-being paradox (Morrison, 2022) is that the negative externalities of large cities on well-being (cost of living, pollution, commuting etc.) overcome the positive ones, like job opportunities, amenities, etc. (Fisher, 1974; Okulicz-Kozaryn, 2015). This interpretation is consistent with the competing theory by Richardson (1973) claiming that higher wages in cities are a compensation for their greater negative externalities and disutility, e.g. congestion, pollution, crime, higher rent.

However, this conclusion is rather disappointing, as it seems to contradict the empirical evidence highlighting increasing inflows of people in large cities.

Recent literature is then increasingly questioning this traditional interpretation. First, there is some evidence documenting that the urban well-being paradox can be the outcome of irrational behaviour or false/misplaced hopes of urban migrants expecting to move to places where their satisfaction is high (Hanell, 2022).^1^ Second, there is more and more interest in understanding the relative role and interplay of people and place characteristics in the explanation of the urban well-being paradox (Burger et al., 2020; Cardoso et al., 2019; Morrison, 2022; Hoogerbrugge & Burger, 2021; Morrison & Weckroth, 2018).

Following this latter group of studies, this paper proposes a contribution to unpacking the urban well-being paradox, conceptually and empirically. The central argument of the paper rests on two premises. First, urban population is inherently heterogeneous and diverse (Florida et al., 2013). Diversity is in fact one of the most distinctive traits of cities, and diversity increases with city size. Importantly, urban residents can be categorised according to multiple dimensions, including some of the individual-level characteristics frequently considered as the determinants of subjective well-being, such as education and income. These categories of individuals, i.e. those with better education, employment opportunities and income, are more likely than the others to grasp the advantages from urbanisation economies in terms of accessibility to diversified and rich labour markets, consumption amenities and highly specialised and customised services (Burger et al., 2020; Cardoso et al., 2019; Morrison, 2022; Hoogerbrugge & Burger, 2021; Morrison & Weckroth, 2018). At the same time, they are the least likely to suffer from the disadvantages of urbanization, in terms of higher costs of living, congestion and lower environmental quality. However, this group of individuals, though primarily located in metropolitan settings, generally represents a minority of urban population (Autor & Dorn, 2013), which is instead unbalanced towards groups of individuals exhibiting characteristics that are negatively associated with subjective well-being (Morrison, 2022). The relative balance of different groups of individuals within cities is likely to be at the origins of the urban well-being paradox (Carlsen & Lekners, 2022). If the majority of the urban population is made of individuals with traits that, generally, are negatively associated with subjective well-being, then the urban well-being paradox is likely to emerge.

Second, urban agglomeration advantages are not spatially bounded but can filter along the urban hierarchy, depending on the distance from more urbanised settings (Lenzi & Perucca, 2018, 2021a). According to the ‘borrowed size’ concept proposed by Alonso (1973), urban externalities are likely to spread out to the surrounding areas, i.e. cities are sources of externalities affecting urban residents (direct effects) as well as of externalities affecting residents of surrounding areas (indirect effects). The intensity and spatial range of such effects are proportional to the characteristics (i.e. size and, thus, functions and rank) of the city generating them. At the same time, the recipient areas will be affected by such urban externalities with different intensities, i.e. depending on their distance from the originating city. Therefore, not only location in but also distance from large cities is likely to matter for individuals’ life satisfaction, and this effect is likely to be greater for those groups of individuals that appreciate the most urbanisation advantages (e.g. educated and affluent ones).

In short, the balance between (negative) direct effects and (positive) indirect effects of urbanisation can be mediated by specific individual characteristics and the relative weight, within recipient cities, of different groups of individuals characterised by traits positively or negatively associated with life satisfaction.

On empirical grounds, the paper makes use of a multilevel modelling framework in order to explore the effect of individual level characteristics in mediating the effect of urbanisation on subjective well-being. Multilevel modelling in fact allows investigating the interplay between variables available at multiple scales of analysis, i.e. the urban and individual level ones (Köppen et al., 2021, Lenzi & Perucca, 2021b). The use of a large dataset on the subjective well-being reported by more than 250,000 EU citizens, sourced from Eurobarometer surveys in the period 2004–2010, makes possible to test empirically the association between subjective well-being, urbanisation and several individual level characteristics as well as of the interplay between individual and urban level variables.

The rest of the paper is organised as follows. Section 2 elaborates the hypotheses to be tested empirically. Section 3 presents data and methods. Section 4 discusses the results and Section 5 concludes with some final remarks.

## The Urban Well-Being Paradox Revisited: The Hypotheses

The literature on the geography of subjective well-being, defined as the extent to which individuals are satisfied with their own life, pointed out the occurrence, at least in developed countries, of an urban/rural divide: people living in the most urbanized regions tend to be significantly less satisfied than people living in rural areas (Okulicz-Kozaryn, 2011). This stylised fact is frequently indicated in the literature as the urban well-being paradox: in spite of improved and more diversified job opportunities, accessibility to a wider range of specialised services and greater consumption amenities, people in (large) cities generally report lower levels of subjective well-being (Morrison, 2022).

The study of the relationship between individuals’ subjective well-being and the typology of their setting of residence, however, overlooked an important conceptual aspect. In fact, cities are the most diverse type of settlement and the diversity of cities has been long considered a key factor behind its enhanced opportunities for individual and aggregate development (Florida et al., 2013; Glaeser, 2011). Diversity in cities concerns not simply economic activities but also individuals; importantly, diversity manifests itself according to multiple dimensions including skills, education, occupation and income. In short, cities are made of individuals who are not alike, and may differently appreciate and be able to enjoy the advantages from urbanisation (e.g. amenities, diversified job markets, accessibility to advanced services) as well as to suffer from its disadvantages (e.g. congestion, pollution, and especially high rent). Recent literature is increasingly highlighting the role of compositional effects and people-related aspects, i.e. individual heterogeneity, in the explanation of the relationship between urbanisation and subjective well-being (Burger et al., 2020; Cardoso et al., 2019; Morrison, 2022; Hoogerbrugge & Burger, 2021; Morrison & Weckroth, 2018).

Characteristics such as the level of education, the type of occupation and, more generally, the income level can mediate the capacity to reap (respectively, mitigate) urbanisation advantages (respectively, disadvantages), and thus affecting individual subjective well-being. There are two main reasons supporting this statement. First, these characteristics are unanimously associated with higher subjective well-being levels (Dolan et al., 2008). Second, cities are the place in which better educated and better paid workers are disproportionately located (and increasingly sort into); cities in fact host more diversified job opportunities with higher salaries, being the place where most affluent people reside (D’Acci, 2019; Castells-Quintana et al., 2020). Several mechanisms concur to this outcome, and are all related to the operation of agglomeration forces in cities, and their increasing strength as city size grows. In large cities, in fact, the skill distribution is especially wide in order to meet the diversified requirements of metropolitan job markets. In particular, agglomeration enables market size to grow, specialisation and, thus, productivity to increase, ultimately leading to greater returns to skills and higher incomes (Duranton, 2019). Moreover, large cities are increasingly innovation hubs and attractive for technology and creative talents, raising more their productivity (and wages) than those of their less talented peers (Behrens & Nicoud, 2014). However, even if highly educated and better-paid individuals are generally disproportionately concentrated in large urban areas, they still represent a minority there; in fact, in cities the proportion of skilled jobs is far lower than that of low skilled ones (Autor & Dorn, 2013).

All this leads to two main conclusions. First, larger cities are the most unequal settings especially in terms of income disparities, suggesting a scaling of inequalities (Castells-Quintana et al., 2020; Glaeser et al., 2015; Sarkar et al., 2016); large cities attract both very skilled and educated individuals earning possibly superstar compensations, as well as individuals at the bottom of the skill and education distribution with unsecure gig-jobs. Spatial inequalities have been recently highlighted as an important source of individual and political discontent (Antonucci et al., 2021; Rodríguez-Pose, 2018). Second, and possibly more importantly from the subjective well-being perspective, education, occupation and income are key determinants of individual life satisfaction. Heterogeneity of individuals in these respects translates first into (income) disparities across people and, second, on opposite effects on subjective well-being (Burger et al., 2020; Cardoso et al., 2019; Morrison, 2022; Hoogerbrugge & Burger, 2021; Morrison & Weckroth, 2018). The final effect on urban subjective well-being depends on the relative size of the different groups of individuals, and can be negative as far as the group of more disadvantaged people experiencing worse or deteriorated living conditions, and thus expressing lower subjective well-being, is larger than the group of privileged ones. With a shortcut, the urban well-being paradox strongly depends on negative distributional effects, which are particularly strong in cities; the larger the proportion of those worse off with respect of the whole population, the larger the inequalities, on the one hand, and the lower the aggregate urban well-being, on the other.

Put more concisely, in cities, there co-exist diverse groups of individuals characterised by different levels of education, job opportunities and earnings, and consequently, perceived life satisfaction. These groups, however, have highly unbalanced sizes, with important consequences on the aggregate perceived level of life satisfaction in cities.

For the minority of highly educated individuals, large cities enable better job opportunities, higher earnings, a rich variety of consumption amenities (Glaeser et al., 2001) and the accessibility to a large spectrum of highly specialised and customised services, not available elsewhere. The positive effect of these enhanced possibilities on their life satisfaction is expected to mitigate the negative impact of urbanization disadvantages on subjective well-being (Burger et al., 2020; Cardoso et al., 2019; Morrison, 2022; Hoogerbrugge and Burger, 2021; Morrison & Weckroth, 2018). Differently, the largest majority of less educated individuals, with inferior job opportunities and limited earnings might find difficult to grasp such advantages and may indeed experience only the downsides of urban life, like congestion, pollution, commuting time and, above all, high rent, with negative consequences on life satisfaction. As far as the latter effect prevails, the outcome is the urban paradox frequently detected in empirical data in a large variety of studies.

Accordingly, the first hypothesis tested in the empirical analysis is:

### H1

The negative effect of urbanisation on individual life satisfaction varies across groups of individuals, and it significantly reduces for more educated and more affluent individuals, facing better job opportunities and life conditions.

This hypothesis well aligns with the most recent findings in the literature on the role of compositional aspects and individual heterogeneity for the explanation of the relationship between urbanisation and subjective well-being. However, it neglects an important aspect. In fact, not only location in, but also distance from large cities is likely to matter for individuals’ life satisfaction. In fact, large cities typically supply the broadest variety of goods and services, while a peripheral location may reflect a poor accessibility to the opportunities and advantages of more urbanised settings. Starting from the seminal contribution by Sirgy and Cornwell (2002), a few papers addressed this issue and have shown that rural towns or villages are associated with higher subjective well-being only when they are embedded in highly urbanized regions (Lenzi and Perucca, 2018). In fact, people do not permanently stay in their city or village. Rather, they commute to neighbouring areas for several reasons, such as the possibility of better (and/or diversified) jobs, improved shopping opportunities, consumption amenities, just to name a few of them. Accordingly, individuals benefit from living close to cities larger than their own, since proximity provides them with potential access to urbanisation benefits not provided in the town of residence, consistently with the idea of ‘borrowed size’ introduced by Alonso (1972) (Hoogerbrugge et al., 2022; Lenzi & Perucca, 2021a, b; Meijers et al., 2016).^2^

However, not all individuals are likely to value to the same extent such proximity, and some individual level characteristics can mediate the relationship between distance from urbanised areas and life satisfaction, i.e. the final balance between ‘borrowed size’ and ‘agglomeration shadows’ can depend on some personal traits. For the same reasons highlighted above, better-educated, more affluent individuals, who enjoy better job opportunities, may show preferences for the top-rank services offered in bigger cities and can appreciate more the opportunity costs of distance and commuting.

Accordingly, the second hypothesis tested in the empirical analysis is:

### H2

Distance from top-rank cities negatively affects individual life satisfaction, especially for highly educated, more affluent individuals, who enjoy better job opportunities.

The next section provides details on the data and the methodology applied to test these hypotheses empirically.

## Data and methods

### Data

The database employed in the empirical analysis is made up of several waves of Eurobarometer (EB) survey studies. A recurrent question concerns the degree of life satisfaction of European Union (EU) citizens: respondents are asked to choose whether they are “very unsatisfied”, “rather unsatisfied”, “rather satisfied” or “very satisfied” with their life. The degree of self-reported life satisfaction is frequently used in the literature as a measure of subjective well-being (see among others Deaton, 2008; Hoogerbrugge and Burger, 2018).

Beside the rich temporal coverage of Eurobarometer surveys, there are conceptual reasons to choose life satisfaction as the main subjective well-being dimension to be examined. In fact, subjective well-being is a more general concept encompassing hedonic, cognitive and eudemonic dimensions (Mouratidis, 2019). The cognitive dimension involves a judgement on the relevance of different types of resources/goods/services for life satisfaction in general (Gärling & Gamble, 2012) and therefore is the most relevant one in the present context, in which the focus is on assessing the relationship between advantages and disadvantages from urbanisation and subjective well-being.

The data set used in the present empirical analysis is based on fifteen EB waves (more than 250,000 observations) between 2004 and 2010.^3^

Specifically, life satisfaction is assumed to depend on a set of individual characteristics and other variables characterizing the context of residence. In details, respondents provide information on their demographic and socioeconomic status (age, gender, education, occupation, marital status), identified by previous literature among the most influential determinants of life satisfaction (Dolan et al., 2008).

Following the discussion presented in Sect. 2, the most interesting variables for the purpose of the present analysis concerns education, occupation and income. Highly educated individuals are those with tertiary educational attainment or above. Individuals with top occupations are professionals, managers and business owners. The identification of high-income individuals, instead, is more complex. Unfortunately, EB surveys do not provide consistent information across waves about the income and/or wealth of the respondents. Therefore, two variables have been included as a proxy for individual income: a dummy variable for apartment ownership and a dummy variable taking value 1 when the respondent reports no difficulties in paying bills/ making ends meet. Higher income individuals are more likely to own an apartment and less likely to have trouble in paying bills.^4^

Importantly, respondents report their NUTS3 region of residence.^5^ This information is particularly relevant to test the association between life satisfaction and the degree of urbanization and the distance from urban settings of higher rank. Differently from most of the existing studies, classifying individuals’ urbanization level of the region of residence, at best, at the NUTS2 level, this paper exploits information at the NUTS3 level, with an important advantage. In fact, in the European context, NUTS3 is the territorial unit that approximates at best urban areas, although defined according to administrative criteria. NUTS3 can therefore be considered as a fair proxy for cities. Moreover, the NUTS3 level captures in a much finer way the degree of urbanisation of the setting in which respondents live. In fact, while within NUTS2 regions urban settings of different kinds may coexist, at the lower level of the nomenclature it is fair to assume that each area is characterised by its own specific positioning in the urban hierarchy. Therefore, even if within each NUTS3, the largest city might not completely cover the surface area, it is, however, fairly reasonable to assume that the rank of the main city in each NUTS3 strongly characterises the functions and markets supplied in the NUTS3 itself. Specifically, urbanization is captured by a set of dummy variables accounting for the size of the largest city in the NUTS3 of residence of the respondent. Urban ranking comes from the official classification of cities provided by Eurostat. According to this definition, EU cities are classified into six mutually exclusive groups, from 1st rank to 6th rank (see Table A3 in Online Appendix). In our analysis, 1st rank (i.e. cities with more than one million inhabitants) and 2nd rank (500 k—1 million inhabitants) cities are grouped in one category.^6^ The distance from top-rank cities (i.e. 1st and 2nd rank cities) is measured in terms of travel time by car (as in Polèse and Sheamur, 2006). Finally, the per capita income in the NUTS3 of residence represents an additional control variable for the overall level of wealth in the area of residence.

The full variables description is reported in the Online Appendix (Table A3), while Table 1 below shows some summary statistics.

**Table 1:** Descriptive statistics of the variables employed in the empirical analysis

*Categorical and dummy variables (individual level, all years 2004–2010 pooled)*	
Life satisfaction	1(very unsatisfied): 6.23%
	2(rather unsatisfied): 18.26%
	3(rather satisfied): 52.83%
	4(very satisfied): 22.68%
Tertiary education	Tertiary education: 35.59%
Apartment ownership	Apartment ownership: 48.02%
No difficulty with bills	No difficulty with bills: 57.17%
Make end meets	Make ends meet: 27.23%
Occupation	Professional/manager and business owner: 4.40%
	Shop owner: 2.73%
	Employee: 27.98%
	Farmer/ fisherman: 1.24%
	House person: 9.24%
	Student: 5.34%
	Retired: 24.88%
	Manual worker: 18.15%
	Unemployed: 6.04%
Female	Female: 54.24%
Marital status	Married: 52.69%
	Single: 29.46%
	Divorced: 7.91%
	Widower: 9.94%
*Continuous variables (individual level, all years 2004–2010 pooled)*	
Age	Age of the respondent: 44.75 (mean) 21.02 (standard deviation)
Children	N. of children in the household: 0.275 (mean) 0.656 (standard deviation)

*City-level variables*			
Per capita GDP		*Mean*	*SD*
	Per capita GDP (2004)	20,480	11,294
	Per capita GDP (2010)	22,984	12,219

City ranking	First-rank: 1.78% of NUTS3 regions		
	Second-rank: 3.40% of NUTS3 regions		
	Third-rank: 7.57% of NUTS3 regions		
	Fourth-rank: 23.49% of NUTS3 regions		
	Fifth-rank: 24.03% of NUTS3 regions		
	Sixth-rank: 39.72% of NUTS3 regions		

Travel time (hours)		Mean	SD
	Distance to closest 1st rank	*4.469*	*3.887*

### Methods

The relationship between life satisfaction and the other characteristics, whose empirical measurement was presented in the previous section, can be formalized as follows:

1 $$LF_{{irct}} = \theta \left( {urbanization_{r} } \right) + \alpha \left( {tertiaryeducation_{{irct}} } \right) + + \beta \left( {occupation_{{irct}} } \right) + \gamma \left( {income_{{irct}} } \right) + \delta X_{{irct}} + \eta Q_{{rt}} + \tau _{t} + \mu _{c} + \rho _{r} + \varepsilon _{{irct}}$$

where *i* stands for the individual, *r* and c respectively for the NUTS3 and country of residence, and *t* for the wave of the survey study. Importantly, EB surveys do not have a panel structure, i.e. it is not possible to observe the same individuals in different periods. Therefore, equation [1] has been estimated on a dataset pooling EB data in the period 2004–2010.^7^

The parameters of interest to be estimated are:

- α, which accounts for tertiary education,
- β, which accounts for the occupation category (i.e. professionals, managers and business owners),
- γ, which accounts for apartment ownership and lack of problems in paying bills,
- θ, which accounts for the urbanisation intensity in the NUTS3 of the respondent’s residence or, alternatively, the travel time distance from top-rank (i.e. 1st and 2nd rank) cities.

Estimates control for individual characteristics (*X*), per capita income in the NUTS3 of residence (*Q*) and survey-specific effects (*τ*).

Importantly, equation [1] has been expanded in order to include interaction effects between the urbanisation variable and the travel time distance from 1st and 2nd rank cities with the individual level education, occupation and income variables. The estimation of these interaction effects provides the test of hypotheses 1 and 2 elaborated in Sect. 2.

The empirical estimation of equation [1] presents a number of methodological issues, related to: *i)* the choice of the most appropriate statistical techniques to be adopted, *ii)* the hierarchical structure of the data.

The literature on subjective well-being makes use of both models for categorical dependent variables and linear models, generally leading to substantially consistent results (Ferre-i-Carbonell and Frijters, 2004). In this study, linear regression analysis is adopted. The use of alternative statistical techniques (i.e. models for categorical variables) does not lead to findings significantly different from those reported in the next section.^8^

The second methodological issue concerns the hierarchical structure of the data. Individuals in the sample are nested within cities (i.e. NUTS3), at the first level, and within countries, at the second one. This hierarchical structure of the data may imply that two randomly selected individuals from the same area are more similar, in terms of life satisfaction, than two people randomly chosen from other groups. If these group-effects are not taken into account, the independence assumption of the residuals from equation [1] does not hold. In order to treat explicitly the hierarchical structure of the data and to adjust standard errors, a multilevel linear random intercept model has been estimated, where the intercept of the group regression lines is allowed to randomly vary across NUTS3. Therefore, in equation [1], $\mu_{c}$ is the effect of country *c*, $\delta_{rc}$ is the effect of the NUTS3 *r* within country *c* and $\varepsilon_{irc}$ is the residual error term.

The next section discusses the results from the estimation of equation [1].

## Results

Table 2 reports the estimates of equation [1]. Variables are classified in two groups, individual and urban characteristics. For both groups, the first variables to be listed (highlighted in bold) are those central to the testing of hypotheses 1 and 2.

**Table 2:** Life satisfaction: the interplay between urbanisation, education, occupation and income

	[a]	[b]	[c]	[d]	[e]	[f]
*Level 1: individual characteristics*						
Tertiary education	0.112***	0.108***	0.112***	0.110***	0.094***	0.105***
	(0.003)	(0.003)	(0.003)	(0.004)	(0.006)	(0.007)
Apartment ownership				0.104***		
				(0.004)		
No difficulty with bills					0.326***	
					(0.006)	
Make ends meet						0.288***
						(0.009)
Professional /manager /business owner	0.358***	0.358***	0.355***	0.338***	0.407***	0.281***
	(0.008)	(0.008)	(0.008)	(0.009)	(0.015)	(0.016)
Shop owner	0.265***	0.265***	0.265***	0.250***	0.354***	0.203***
	(0.009)	(0.009)	(0.009)	(0.010)	(0.017)	(0.019)
Employee	0.282***	0.282***	0.282***	0.270***	0.363***	0.208***
	(0.005)	(0.005)	(0.005)	(0.005)	(0.010)	(0.009)
Farmer/ fisherman	0.154***	0.153***	0.154***	0.130***	0.244***	0.111***
	(0.013)	(0.013)	(0.013)	(0.015)	(0.027)	(0.028)
Manual worker	0.162***	0.162***	0.162***	0.147***	0.272***	0.104***
	(0.005)	(0.005)	(0.005)	(0.006)	(0.011)	(0.011)
Houseperson	0.111***	0.111***	0.111***	0.087***	0.217***	0.037***
	(0.005)	(0.005)	(0.005)	(0.006)	(0.014)	(0.009)
Student	0.273***	0.272***	0.273***	0.225***	0.412***	0.192***
	(0.008)	(0.008)	(0.008)	(0.009)	(0.015)	(0.017)
Retired	0.145***	0.145***	0.145***	0.127***	0.221***	0.087***
	(0.005)	(0.005)	(0.005)	(0.006)	(0.011)	(0.011)
Age	−0.024***	−0.024***	−0.024***	−0.026***	−0.019***	−0.026***
	(0.001)	(0.001)	(0.001)	(0.001)	(0.001)	(0.001)
Age^2	0.000***	0.000***	0.000***	0.000***	0.000***	0.000***
	(0.000)	(0.000)	(0.000)	(0.000)	(0.000)	(0.000)
Female	0.014***	0.014***	0.014***	0.016***	0.022***	0.017***
	(0.003)	(0.003)	(0.003)	(0.003)	(0.005)	(0.006)
Children	0.002	0.002	0.002	0.005**	0.019***	−0.001
	(0.002)	(0.002)	(0.002)	(0.003)	(0.004)	(0.005)
Single	−0.118***	−0.118***	−0.118***	−0.116***	−0.073***	−0.111***
	(0.004)	(0.004)	(0.004)	(0.004)	(0.006)	(0.009)
Divorced	−0.255***	−0.255***	−0.255***	−0.236***	−0.180***	−0.230***
	(0.005)	(0.005)	(0.005)	(0.006)	(0.010)	(0.011)
Widower	−0.208***	−0.208***	−0.208***	−0.200***	−0.152***	−0.197***
	(0.005)	(0.005)	(0.005)	(0.006)	(0.010)	(0.011)
*Level 2: regional characteristics*						
GDP region	0.021***	0.021***	0.021***	0.017***	0.003	0.001
	(0.006)	(0.006)	(0.006)	(0.007)	(0.009)	(0.010)
5th rank cities	−0.026**	−0.026**	−0.026**	−0.019	0.003	−0.029
	(0.012)	(0.012)	(0.012)	(0.013)	(0.017)	(0.019)
4th rank cities	−0.010	−0.010	−0.010	−0.005	0.012	−0.041**
	(0.013)	(0.013)	(0.013)	(0.014)	(0.018)	(0.020)
3rd rank cities	−0.009	−0.009	−0.009	0.006	0.016	0.002
	(0.019)	(0.019)	(0.019)	(0.019)	(0.024)	(0.027)
1st and 2nd rank cities	−0.052**	−0.058***	−0.052**	−0.030	−0.053*	−0.054*
	(0.020)	(0.021)	(0.020)	(0.021)	(0.028)	(0.030)
1st and 2nd rank * tertiary education		0.018**				
		(0.007)				
1st and 2nd rank * Professional /manager /business owner			0.001			
			(0.015)			
1st and 2nd rank * apartment owner				−0.009		
				(0.009)		
1st and 2nd rank * no difficulty with bills					0.326***	
					(0.006)	
1st and 2nd rank * make ends meet						0.046**
						(0.019)
EB wave dummies	Yes	Yes	Yes	Yes	Yes	Yes
Constant	3.301***	3.302***	3.301***	3.338***	2.912***	3.344***
	(0.090)	(0.090)	(0.090)	(0.096)	(0.088)	(0.088)
Random effects						
Level 1 (individual) variance	0.442	0.442	0.442	0.436	0.418	0.423
	(0.001)	(0.001)	(0.001)	(0.001)	(0.002)	(0.003)
Level 2 (region) variance	0.011	0.011	0.011	0.011	0.014	0.018
	(0.001)	(0.001)	(0.001)	(0.001)	(0.001)	(0.002)
Level 3 (country) variance	0.141	0.141	0.141	0.16	0.123	0.121
	(0.047)	(0.047)	(0.047)	(0.054)	(0.041)	(0.041)
ICC – level 2 (region)	0.019	0.019	0.019	0.018	0.025	0.032
ICC – level 3 (country)	0.237	0.237	0.237	0.264	0.222	0.216
Observations	252,271	252,271	252,271	186,066	68,315	50,799

Reference categories: unemployed (occupation), married (marital status), 6th rank cities (city rank)

Standard errors in parentheses. *** p < 0.01, ** p < 0.05, * p < 0.1

Moving directly to the most relevant individual level variables, results indicate that tertiary educated individuals report higher levels of life satisfaction. Similarly, affluent individuals, with apartment ownership and without difficulties in paying bills, experience higher well-being.^9^ Similar conclusions apply to individuals with professional, managerial or entrepreneurial occupations.

Living in 1st or 2nd rank NUTS3 dampens life satisfaction, as expected. This negative effect of urbanisation, however, is limited to NUTS3 with very large cities (above 500,000 inhabitants) and vanishes in smaller cities (i.e. 3rd rank and lower), which generally do not report any significant effect.^10^

These results are consistent with the literature. What is new, instead, are the results on the interaction effects, which are consistent with the expectations discussed in Sect. 2. The negative effect of urbanisation on life satisfaction, in fact, reverses for educated and affluent people. The interaction terms are positive and significant, while the coefficients of the non-interacted variables preserve their sign and significance. In particular, this holds true for tertiary education and for those respondents who make ends meet/ did not have any difficulty in paying their bills.^11^ These findings suggest that educated and affluent people with better occupational prospects experience higher well-being in highly urbanized settings than elsewhere. Moreover, they experience higher levels of well-being than their less educated and affluent peers, regardless their location, since the non-interacted coefficients remain statistically significant. In sum, these results confirm hypothesis 1.

For what concerns the individual level control variables, they all present the expected sign and significant. Age has a U-shaped effect on life satisfaction; all occupations and employment status are associated with a positive effect on life satisfaction with respect to unemployment. Female are generally more satisfied with life as well as individuals with a partner with respect to the other marital statuses. Finally, income per capita in the NUTS3 region of residence is positively associated with higher life satisfaction.

**Fig. 1:**
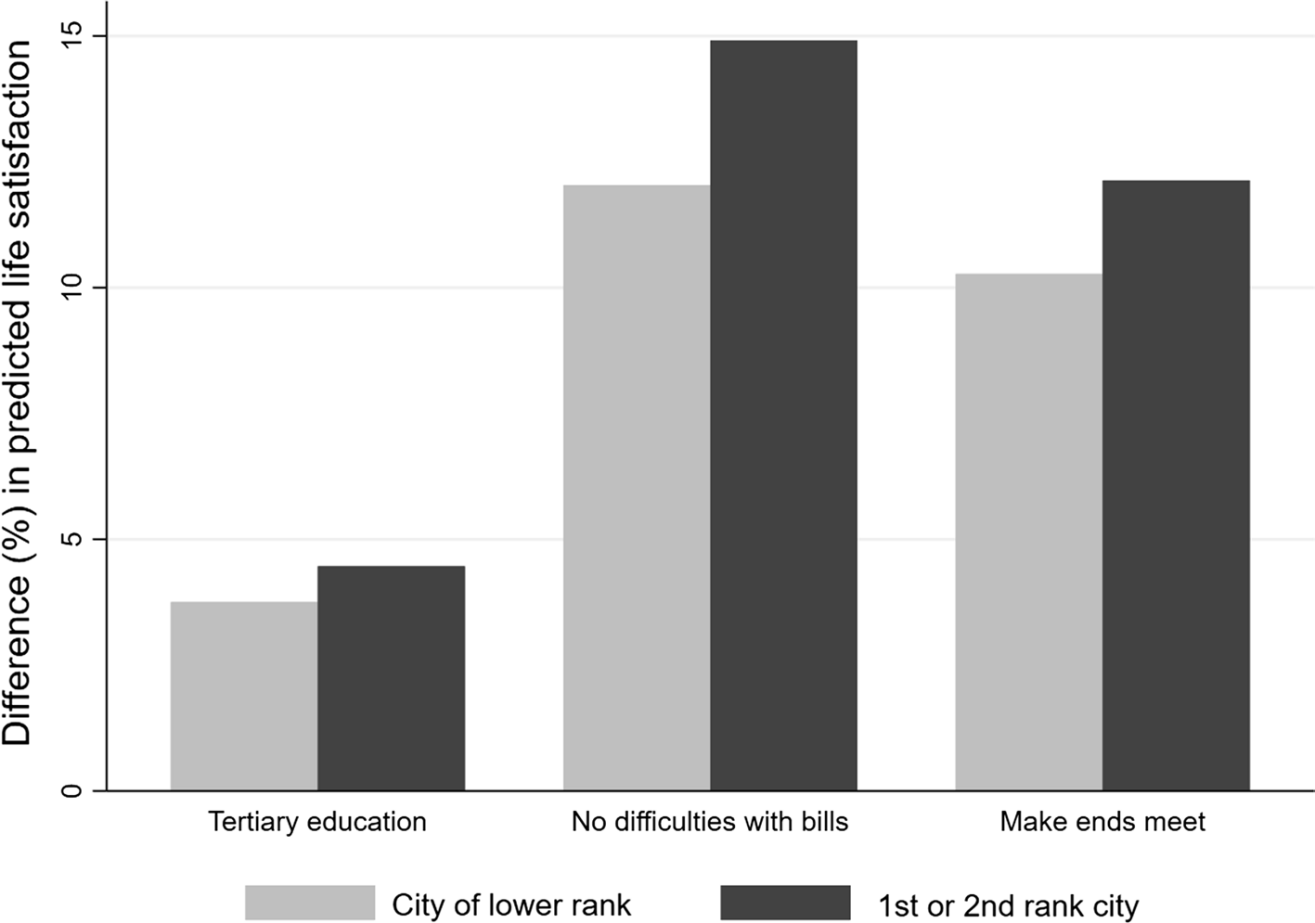
Difference (%) in the predicted life satisfaction of people in condition of relative advantage compared with the others, in 1st and 2nd rank NUTS3 vs less urbanized areas

The results of the significant interactions are graphically represented in Fig. 1. The figure shows the percentage difference in predicted life satisfaction between the individuals with (respectively, without) each of the conditions of personal advantage. This difference is estimated for both those living in 1st or 2nd rank NUTS3 (the dark grey bars in Fig. 1) and those living in cities of lower rank (the light grey bars). For instance, the figure shows that, in 1st and 2nd rank NUTS3, keeping other things constant, the predicted life satisfaction of respondents with tertiary education is 4.47 per cent higher than those with lower education. In cities of lower rank, however, the relative “premium” of tertiary education is 3.75 per cent. Consistently with the output reported in Table 2, the same applies to the other conditions of personal advantage. In 1st and 2nd rank NUTS3, being able to pay one’s own bills/ make ends meet leads to a predicted level of life satisfaction of about 14.91 (no difficulty with bills) and 12.31 per cent (make ends meet) higher than the others. In less urbanized areas, these figures reduce respectively to 12.03 and 12.13 per cent.

**Fig. 2:**
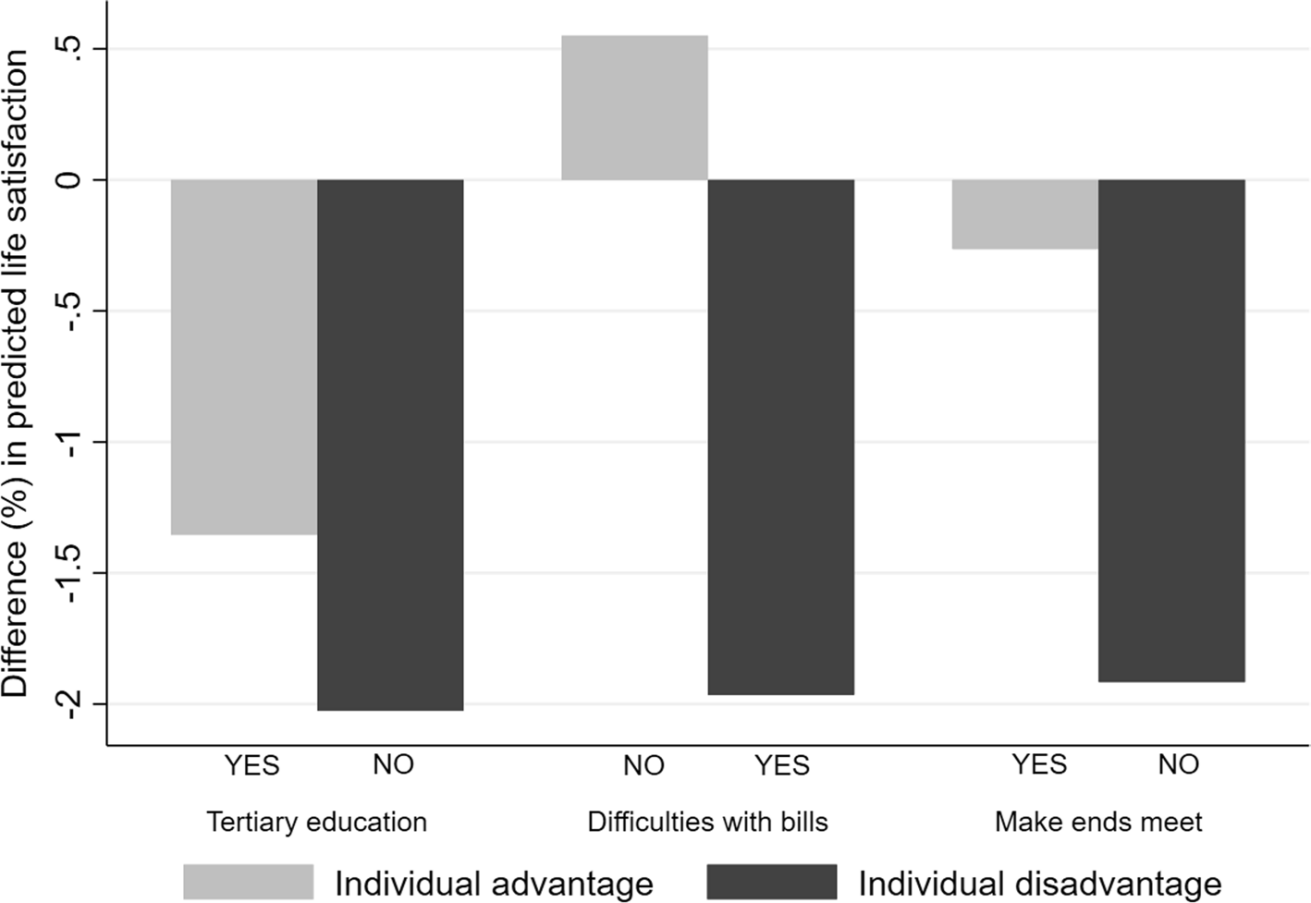
Difference (%) in the predicted life satisfaction of people living in 1st and 2nd rank NUTS3 vs less urbanized regions: respondents in condition of individual advantage and disadvantage

A similar reasoning can be applied by comparing the predicted life satisfaction of individuals with similar characteristics, but living in areas with different levels of urbanization (Fig. 2). This exercise is useful for understanding whether the negative association between life satisfaction and living in large cities (i.e. the coefficient of the “1st and 2nd rank cities” dummy in Table 1, column [a]) vanishes for people in conditions of educational and economic advantage.

Figure 2 reports the difference (in percentage) of the average life satisfaction reported in 1st and 2nd rank cities compared with lower rank ones, for individuals in conditions of, respectively, relative advantage (the light grey bars) and relative disadvantage (the dark grey bars).

The figure shows, for instance, that individuals with tertiary education are, in 1st and 2nd rank cities, on average 1.35 per cent less satisfied than tertiary educated people living in cities of lower rank. On the other hand, people with secondary (or lower) education in 1st and 2nd rank cities are on average 2.03 per cent less satisfied than their counterparts living in smaller urban areas. Therefore, in this case, higher education does not fully compensate the negative effect of living in a highly urbanized area. The opposite holds when considering the two variables on the individuals’ capability of coping with their expenses. Figure 2 shows that, if we consider those who do not have difficulties in paying the bills or in making end meets, the predicted life satisfaction of 1st and 2nd rank cities’ residents is basically equivalent (between ± 0.6 per cent) to the life satisfaction of the same typology of respondents living in less urbanized areas. Rather, individuals who experience difficulties in paying their bills or making end meets are significantly less satisfied in large cities than in areas with less intense urbanization.

These findings suggest that the average negative coefficient associated with 1st and 2nd rank cities hides strong disparities in well-being, between those who can afford (and therefore enjoy) the advantages of urbanization and those who cannot. Moreover, the different conditions of individual advantage (disadvantage) often correlate, so that their positive (negative) effect cumulates. For instance, people with difficulties in paying the bills are also more likely to have poor education, to be in a precarious job condition, etc. While the present paper is not aimed at disentangling and measuring these cumulative effects, the topic is highly interesting and still relatively understudied in the literature on subjective well-being.

Moving to the effects of distance from the closest 1st or 2nd rank NUTS3 (Table 3), results are largely consistent with those of Table 2.^12^ By construction, residents in 1st and 2nd rank NUTS3 are excluded from the analysis.^13^ Also in this set of estimates, tertiary educated individuals report, on average, higher levels of life satisfaction as well as affluent individuals, those with apartment ownership and without difficulties in paying bills / making end meets. The same conclusions hold for individuals with professional, managerial or entrepreneurial occupations.

**Table 3:** Distance from 1st rank cities and life satisfaction: the role of education, occupation and income

	[a]	[a1]	[a2]	[a3]	[a4]	[b1]	[c1]	[d1]	[e1]	[f1]
	Distance band from the closest 1st rank NUTS3									
	Whole sample	< 0.5 h	0.5—1.5 h	1.5—3 h	> 3 h	Whole sample	Whole sample	Whole sample	Whole sample	Whole sample
*Level 1: individual characteristics*										
Tertiary education	0.108***	0.152***	0.111***	0.139***	0.105***	0.106***	0.108***	0.109***	0.097***	0.102***
	(0.004)	(0.022)	(0.009)	(0.010)	(0.008)	(0.005)	(0.004)	(0.004)	(0.007)	(0.008)
Apartment ownership								0.114***		
								(0.005)		
No difficulty with bills									0.345***	
									(0.008)	
Make ends meet										0.303***
										(0.013)
Professional /manager and business owner	0.362***	0.396***	0.337***	0.422***	0.326***	0.362***	0.362***	0.341***	0.424***	0.280***
	(0.009)	(0.048)	(0.021)	(0.021)	(0.019)	(0.009)	(0.011)	(0.010)	(0.017)	(0.019)
Shop owner	0.264***	0.279***	0.257***	0.365***	0.229***	0.264***	0.264***	0.249***	0.369***	0.197***
	(0.010)	(0.084)	(0.023)	(0.024)	(0.019)	(0.010)	(0.010)	(0.012)	(0.019)	(0.021)
Employee	0.287***	0.323***	0.281***	0.337***	0.255***	0.287***	0.287***	0.277***	0.369***	0.218***
	(0.005)	(0.035)	(0.013)	(0.012)	(0.011)	(0.005)	(0.005)	(0.006)	(0.011)	(0.010)
Farmer/ fisherman	0.149***	0.354	0.162***	0.234***	0.069***	0.149***	0.149***	0.127***	0.235***	0.101***
	(0.013)	(0.440)	(0.046)	(0.035)	(0.021)	(0.013)	(0.013)	(0.015)	(0.027)	(0.029)
Manual worker	0.169***	0.185***	0.191***	0.181***	0.151***	0.169***	0.169***	0.155***	0.279***	0.110***
	(0.006)	(0.046)	(0.015)	(0.013)	(0.012)	(0.006)	(0.006)	(0.007)	(0.012)	(0.012)
Houseperson	0.111***	0.180***	0.093***	0.174***	0.080***	0.111***	0.111***	0.088***	0.216***	0.042***
	(0.006)	(0.039)	(0.014)	(0.013)	(0.012)	(0.006)	(0.006)	(0.006)	(0.015)	(0.010)
Student	0.279***	0.239***	0.301***	0.361***	0.246***	0.279***	0.279***	0.238***	0.422***	0.205***
	(0.009)	(0.057)	(0.022)	(0.022)	(0.017)	(0.009)	(0.009)	(0.010)	(0.016)	(0.020)
Retired	0.147***	0.218***	0.141***	0.144***	0.112***	0.147***	0.147***	0.130***	0.221***	0.092***
	(0.006)	(0.040)	(0.015)	(0.014)	(0.012)	(0.006)	(0.006)	(0.007)	(0.012)	(0.012)
Age	-0.023***	-0.028***	-0.022***	-0.025***	-0.021***	-0.023***	-0.023***	-0.025***	-0.019***	-0.025***
	(0.001)	(0.004)	(0.001)	(0.001)	(0.001)	(0.001)	(0.001)	(0.001)	(0.001)	(0.001)
Age^2	0.000***	0.000***	0.000***	0.000***	0.000***	0.000***	0.000***	0.000***	0.000***	0.000***
	(0.000)	(0.000)	(0.000)	(0.000)	(0.000)	(0.000)	(0.000)	(0.000)	(0.000)	(0.000)
Female	0.012***	0.066***	0.018**	-0.008	0.017***	0.012***	0.012***	0.015***	0.020***	0.015**
	(0.003)	(0.020)	(0.008)	(0.007)	(0.007)	(0.003)	(0.003)	(0.004)	(0.006)	(0.007)
Children	0.002	-0.001	0.003	-0.013**	-0.005	0.003	0.002	0.002	0.005*	0.019***
	(0.002)	(0.016)	(0.006)	(0.006)	(0.005)	(0.002)	(0.002)	(0.002)	(0.003)	(0.004)
Single	-0.117***	-0.207***	-0.114***	-0.104***	-0.117***	-0.118***	-0.117***	-0.117***	-0.114***	-0.075***
	(0.004)	(0.027)	(0.010)	(0.010)	(0.009)	(0.004)	(0.004)	(0.004)	(0.005)	(0.007)
Divorced	-0.262***	-0.352***	-0.306***	-0.265***	-0.228***	-0.256***	-0.262***	-0.262***	-0.245***	-0.182***
	(0.006)	(0.034)	(0.014)	(0.013)	(0.013)	(0.005)	(0.006)	(0.006)	(0.007)	(0.011)
Widower	-0.209***	-0.296***	-0.259***	-0.189***	-0.172***	-0.210***	-0.209***	-0.209***	-0.202***	-0.154***
	0.002	-0.001	0.003	-0.013**	-0.005	(0.005)	(0.006)	(0.006)	(0.007)	(0.011)
Level 2: regional characteristics										
GDP region	0.032***	0.043***	0.068***	0.017	0.091***	0.022***	0.032***	0.032***	0.023***	0.014
	(0.007)	(0.015)	(0.017)	(0.019)	(0.025)	(0.007)	(0.007)	(0.007)	(0.008)	(0.011)
5th rank cities	-0.030**	-0.019	-0.055**	-0.035	-0.010	-0.028**	-0.030**	-0.030**	-0.021	-0.002
	(0.012)	(0.054)	(0.027)	(0.029)	(0.031)	(0.012)	(0.012)	(0.012)	(0.013)	(0.017)
4th rank cities	-0.015	-0.013	-0.098***	0.004	-0.008	-0.011	-0.014	-0.015	-0.006	0.007
	(0.013)	(0.060)	(0.031)	(0.031)	(0.033)	(0.013)	(0.013)	(0.013)	(0.014)	(0.018)
3rd rank cities	-0.013	-0.010	-0.144***	-0.006	-0.004	-0.010	-0.013	-0.013	0.006	0.012
	(0.019)	(0.073)	(0.048)	(0.043)	(0.040)	(0.019)	(0.019)	(0.019)	(0.020)	(0.025)
Distance to closest 1st & 2nd rank	-0.005***	-0.520	-0.099**	-0.067**	-0.006***	-0.005***	-0.005***	-0.003	0.000	0.001
	(0.002)	(0.370)	(0.040)	(0.026)	(0.002)	(0.002)	(0.002)	(0.002)	(0.003)	(0.003)
Distance 1st & 2nd rank * tertiary education						0.001				
						(0.001)				
Distance 1st & 2nd rank * Professional /manager /business owner							0.000			
							(0.003)			
Distance 1st & 2nd rank * apartment owner								-0.005***		
								(0.001)		
Distance 1st & 2nd rank * no difficulty with bills									-0.009***	
									(0.002)	
Distance 1st & 2nd rank * make ends meet										-0.008**
										(0.004)
EB wave dummies	Yes	Yes	Yes	Yes	Yes	Yes	Yes	Yes	Yes	Yes
Constant	3.302***	3.612***	3.283***	3.342***	3.331***	3.303***	3.302***	3.324***	2.908***	3.311***
	(0.089)	(0.214)	(0.123)	(0.125)	(0.120)	(0.089)	(0.089)	(0.096)	(0.088)	(0.089)
Random effects										
Level 1 (individual) variance	0.441	0.380	0.451	0.480	0.456	0.442	0.441	0.436	0.416	0.423
	(0.046)	(0.008)	(0.003)	(0.003)	(0.003)	(0.001)	(0.001)	(0.002)	(0.003)	(0.003)
Level 2 (region) variance	0.011	0.005	0.012	0.012	0.012	0.011	0.011	0.011	0.015	0.019
	(0.001)	(0.003)	(0.002)	(0.002)	(0.002)	(0.001)	(0.001)	(0.001)	(0.001)	(0.002)
Level 3 (country) variance	0.137	0.082	0.119	0.112	0.141	0.137	0.137	0.158	0.118	0.118
	(0.046)	(0.051)	(0.053)	(0.050)	(0.062)	(0.046)	(0.046)	(0.053)	(0.040)	(0.040)
ICC – level 2 (region)	0.019	0.011	0.020	0.019	0.019	0.019	0.019	0.019	0.027	0.034
ICC – level 3 (country)	0.233	0.175	0.204	0.186	0.232	0.233	0.233	0.261	0.216	0.210
Observations	204,316	4,226	34,527	40,535	48,289	204,316	204,316	150,542	54,777	40,783

Reference categories: unemployed (occupation), married (marital status), 6th rank cities (city rank)

Standard errors in parentheses. *** p < 0.01, ** p < 0.05, * p < 0.1

Notice that our measure of travel time distance is expressed as a continuous variable. This may pose an issue if we assume distance decay for the range of urbanization externalities. Put differently, beyond a certain distance-threshold people could be indifferent about the existence of a large city, because the cost (for instance in terms of time, travel costs, etc.) of exploiting its urbanization economies becomes too high. This effect was tested in columns [a1]-[a4] in Table 3. Compared with column [a], where the whole sample is analysed, the estimates reported moving rightwards in Table 3 consider subsamples of the individuals, defined according to different intervals of travel time distance from the closest 1st or 2nd rank NUTS3. Column [a1], for instance, considers only individuals living within 30 min of distance from the closest 1st or 2nd rank. The coefficient associated with distance is not statistically significant. This suggests that, when living close to a large urban area, a further increase in distance does not significantly affect individuals’ life satisfaction. This result is consistent with previous literature. Lenzi and Perucca (2018) found that, within highly urbanized NUTS2 regions (which generally include several NUTS3 areas), people living in rural settings are, other things constant, more satisfied than those living in the main city. If we increase the distance band, for instance considering people living between 0.5 and 1.5 h (column [a2]) and between 1.5 and 3 h (column [a3]) the coefficient of distance becomes negative and statistically significant. Therefore, for more remote locations (relative to the closest 1st or 2nd rank), a further increase in distance is associated with a significantly lower level of life satisfaction, presumably indicating a higher and higher cost of accessing and exploiting urbanization economies. Finally, if we consider individuals living beyond the threshold of 3 h (column [a4]) the negative effect of a further increase in distance on satisfaction does not vanish, but the size of the coefficient noticeably shrinks.^14^ In this latter case, distance is assumed to capture extreme remoteness and the impossibility (due to the high cost associated with travelling) to catch any of the advantages typical of large cities.

Summing up, the results reported in Table 3 show that on average, distance from largest cities dampens life satisfaction, as expected and suggested by the literature (Lenzi & Perucca, 2021a).^15^ Whether this effect differs across alternative categories of individuals is tested in Table 3 (columns b[1]-f[1]). In principle, this negative effect is expected to be especially high for affluent individuals, which suffer more distance from centres offering more diversified job opportunities, consumption amenities and accessibility to advanced services. The findings shown in Table 3 are consistent with hypothesis 2, as the interaction effects between individual level variables and the distance variable are negative and significant with the exception of the occupation dummy and tertiary education. Importantly, the coefficients of the non-interacted variables preserve their sign and significance. Overall, these findings suggest that affluent people experience higher well-being when they live close to large setting. As noted for Table 2, they experience higher levels of well-being than their peers in condition of economic disadvantage, regardless their location. In sum, these results confirm hypothesis 2 and suggest that ‘borrowed size’ effects more than compensate ‘agglomeration shadow’ effects in general, and especially for more affluent individuals. For what concerns the individual and urban level control variables, results are substantially unaltered.

## Conclusions

This paper studied the occurrence of the so-called urban/rural paradox in life satisfaction and its relationship with a selection of individual characteristics, related to the socioeconomic status of the respondents. While our findings confirmed the occurrence of a generalized lower level of life satisfaction in largest cities, they pointed out that this negative effect asymmetrically affects different categories of the population. In particular, a relative better socioeconomic condition, in terms of higher education and income, substantially compensate the negative effect of urbanization on subjective well-being. On the other hand, those affected the most by urbanization diseconomies are the groups of people in condition of socioeconomic disadvantage, whose wellbeing is (other things constant) significantly higher in less urbanized settings.

Our interpretation of this result is that better job opportunities, salaries and all the other advantages of urbanization compensate, for affluent individuals, the disadvantages typical of large cities. Individuals with lower socioeconomic status, on the other hand, experience mostly the costs of urbanization, while they have a limited access to the advantages and opportunities typical of large cities.

These findings convey important policy implications. They suggest that the disparity in subjective well-being is particularly high and intense in large cities. The latter are the place where the individual socioeconomic status plays a strongest role in determining one’s own perceived life satisfaction. While a recent literature pointed to the rapid increase of income and social inequalities in cities over the last two decades (Florida, 2017; Sampson, 2019), the way in which they translate into subjective well-being is still mostly unknown and understudied, even if empirical evidence is expanding in vey recent years (Burger et al., 2020; Cardoso et al., 2019; Morrison, 2022; Hoogerbrugge and Burger, 2021; Morrison & Weckroth, 2018). Individual characteristics matter not only for understanding the relationship between urbanisation and subjective well-being, but also for shedding light on the relationship between ‘borrowed size’ and subjective well-being. In this respect, our findings suggest that proximity to large cities. i.e. ‘borrowed size’ effect, can be a general advantage for everyone. In particular, people with a lower socioeconomic status can work in a large city but live in smaller and cheaper place. Nonetheless, this advantage is again grasped especially by more affluent individuals, suggesting that not only agglomeration per se but also ‘borrowed size’ effects are a sort of privilege for this group of people.

This conclusion warns against blind and simplistic interpretations of the long dominant narrative highlighting the advantages of urbanisation and large cities. The interplay between individual traits and place characteristics largely explains who can benefit from the opportunities disproportionately offered in agglomerated settings and who suffer the most the costs generated by such settings. Importantly, the capacity to reap agglomeration advantages benefit further affluent and educated individuals, thus amplifying existing inequalities and potentially transforming agglomeration externalities into a privilege for élite people.

As one of the first attempt, to the best of our knowledge, to address this issue, our paper showed that urbanization is associated with the strongest disparity in life satisfaction between groups with different socioeconomic status. If, as stated above and extensively discussed in Sect. 2, economic theory explains the occurrence of this imbalance, it is interesting to reflect about the consequences of such phenomenon. Several studies, for instance, recently suggested that the rising of populist, anti-system parties is mostly due to the discontent originated by socioeconomic disparities (McCann, 2020; Rodríguez-Pose, 2018). These hypotheses call for an empirical validation based on the further deepening of the analysis on subjective well-being and its relationship with socioeconomic disparities.

Moreover, the paper compared the well-being of two main groups of individual (1) the higher educated more affluent individuals with a higher socioeconomic status and (2) the group with a lower socioeconomic status. This choice is overall consistent with the majority of works on the subject (Burger et al., 2020; Cardoso et al., 2019; Morrison, 2022; Hoogerbrugge and Burger, 2021; Morrison & Weckroth, 2018). However, this approach overlooks intermediate categories and groups of individuals, for which some literature highlights a considerable deterioration of job opportunities and, thus, subjective well-being (Autor & Dorn, 2013). This is definitively an interesting research direction to pursue in the future.

Lastly, the extension of this analysis to more recent years could help capturing the effects of the widespread diffusion of remote working practices for higher educated people, pushed by the COVID-19 pandemic. The diffusion of remote working is certainly redefining, if not reducing, the desirability of proximity to cities for job purposes.

We do hope to expand our future research in these promising directions.

## Supplementary Information

Below is the link to the electronic supplementary material.

Supplementary file1 (DOCX 41 kb)

## Footnotes

1 We thank an anonymous referee for drawing our attention on this interpretation.

2 In this paper, we follow the approach by Meijers et al., (2016, p.185) proposing that “agglomeration shadows and borrowed size effects are different sides of the same medal called ‘network externalities’. A positive influence of network connectivity on the presence of urbanization economies is referred to as ‘borrowed size’, whereas a negative influence of network connectivity leads to ‘agglomeration shadows’.”.

3 The full list is available in Online Appendix, Table A1. We are aware that the inclusion of more recent Eurobarometer waves could help capturing the effects of the widespread diffusion of remote working practices for higher educated people, pushed by the COVID-19 pandemic. The diffusion of remote working is certainly redefining if not reducing the desirability of proximity to cities for job purposes. Unfortunately, the extension to more recent years is not feasible due to the lack of Eurobarometer data at NUTS3 level after 2010.

4 It is worth noting that these measures of one’s own socioeconomic status are not independent from each other. For instance, higher education is generally associated with less difficulties in paying the bills. Evidence on the strength of these associations is reported in the Online Appendix, Table A2. As shown in Table A2, the intensity of the association between our variables of socioeconomic status is generally positive, but far from perfect. The choice of including several indicators of individual wealth, instead for instance of a summative one, is based on two reasons. First, each of them captures a specific dimension of individual socioeconomic status (educational, professional, and economic), which only partially overlaps with the others (Table A2). Second, Eurobarometer surveys provide no data on respondents’ income and wealth, which has to be indirectly approximated by using information on other individual characteristics. Since each of these proxies is by definition an imperfect measure of (unobserved) income, the contextual use of several indicators allows checking for the robustness of the association between life satisfaction and socioeconomic status.

5 Data at the NUTS3 level are not available for the following countries: UK, Finland, Poland, Czech Republic, Estonia, Lithuania and Slovakia. These countries are therefore excluded from the analysis.

6 Our estimates are also replicated on 1st rank regions only. Results (reported in Table A4 in the Online Appendix) are fully comparable with those reported in the main paper. Notice that, apart from providing evidence on the robustness of our findings, considering 1st rank NUTS3 regions in a separate category is interesting, since cities over 1 million inhabitants produce urban externalities (both positive and negative) whose intensity and typology is significantly different from those supplied in cities of lower size.

7 The coefficient *τ*_*t*_ refers to a set of dummy variables, each accounting for a different survey wave employed in the analysis.

8 Evidence on this is available from the authors upon request.

9 Notice that the variables labelled in Table 2 as “No difficulty with bills” and “Make ends meet” are conceptually very similar, but treated separately due to some differences in Eurobarometer’s question wording. More specifically, in the former case, individuals were asked if “During the last twelve months, would you say you had difficulties to pay your bills at the end of the month?”. In the latter case, respondents had to choose an answer out of four options (from totally disagree to totally agree) to the following question: “Please tell me to what extent you agree or disagree with the following statement: You have difficulties paying all your bills at the end of the month”. These two questions are occasionally (and never simultaneously) included in the surveys, which explains the lower number of observations in columns [e] and [f] of Table 2.

10 NUTS3 regions with 5th rank cities represent an unexpected exception to this general pattern.

11 It is worth mentioning that apartment ownership is significantly associated with higher life satisfaction when comparing 1st rank cities alone with the other kinds of settings. This finding mirrors the higher, and constantly increasing over the last decades, urban rent of major cities. Similarly, also the occupation as managers and professionals is associated with a significantly higher life satisfaction in 1st rank cities, presumably due to the better job opportunities and salaries typical of the largest urban areas. Taken together, these results highlights once more the hierarchical structure of urban systems, which highlights the highly differentiated impact of urbanization economies and diseconomies on individuals’ wellbeing. Evidence on this is reported in Table A4.

12 As for the results reported in Table 2, we provide evidence on the comparison between 1st rank NUTS3 and all the other kinds of urban settings in the Online Appendix, Table A5.

13 EB data does not allow for the localization of individuals within NUTS3. Therefore, it is not possible to know whether they live inside or outside the city.

14 The 95 per cent confidence interval of the coefficient associated with distances above 3 h of travel time from the closest 1st or 2nd rank city does not overlap the intervals of the coefficients associated with distances between 30 min.-1.5 h and 1.5–3 h.

15 Notice that, given the conceptualization of the different urban functions hosted in cities of different size, we could assume the distance from 1st and 2nd rank NUTS3 to affect in particular the life satisfaction of individuals living in areas at the bottom of our ranking. Our empirical findings, not included in Table 3, show that the association between distance from cities of higher rank and individuals’ life satisfaction does not significantly vary across NUTS3 regions with different levels of urbanization. Results on this are in Table A6 of the Online Appendix.
