# Supplementary material for: No Place for Poor Men: On the Asymmetric Effect of Urbanization on Life Satisfaction
Source: Soc Indic Res. 2022 May 28;164(1):165–87. doi: 10.1007/s11205-022-02946-1 (PMC9146818; doi:10.1007/s11205-022-02946-1)
Supplement: Supplementary file 1 — Supplementary file1 (DOCX 41 kb) [file 11205_2022_2946_MOESM1_ESM.docx]

**Appendix**

*Table A1. List of Eurobarometer surveys used in the paper*

European Commission (2012): Eurobarometer 62.0 (Oct-Nov 2004). TNS OPINION & SOCIAL, Brussels [Producer]. GESIS Data Archive, Cologne. ZA4229 Data file Version 1.1.0, doi: 10.4232/1.10962

European Commission (2012): Eurobarometer 62.2 (Nov-Dec 2004). TNS OPINION & SOCIAL, Brussels [Producer]. GESIS Data Archive, Cologne. ZA4231 Data file Version 1.1.0, doi: 10.4232/1.10964

European Commission (2012): Eurobarometer 63.4 (May-Jun 2005). TNS OPINION & SOCIAL, Brussels [Producer]. GESIS Data Archive, Cologne. ZA4411 Data file Version 1.1.0, doi:10.4232/1.10968

European Commission (2012): Eurobarometer 64.2 (Oct-Nov 2005). TNS OPINION & SOCIAL, Brussels [Producer]. GESIS Data Archive, Cologne. ZA4414 Data file Version 1.1.0, doi:10.4232/1.10970

European Commission (2012): Eurobarometer 65.2 (Mar-May 2006). TNS OPINION & SOCIAL, Brussels [Producer]. GESIS Data Archive, Cologne. ZA4506 Data file Version 1.0.1, doi:10.4232/1.10974

European Commission (2012): Eurobarometer 66.1 (Sep-Oct 2006). TNS OPINION & SOCIAL, Brussels [Producer]. GESIS Data Archive, Cologne. ZA4526 Data file Version 1.0.1, doi:10.4232/1.10980

European Commission (2012): Eurobarometer 67.2 (Apr-May 2007). TNS OPINION & SOCIAL, Brussels [Producer]. GESIS Data Archive, Cologne. ZA4530 Data file Version 2.1.0, doi:10.4232/1.10984

European Commission (2012): Eurobarometer 68.1 (Sep-Nov 2007). TNS OPINION & SOCIAL, Brussels [Producer]. GESIS Data Archive, Cologne. ZA4565 Data file Version 4.0.1, doi:10.4232/1.10988

European Commission (2013): Eurobarometer 69.2 (Mar-May 2008). TNS OPINION & SOCIAL, Brussels [Producer]. GESIS Data Archive, Cologne. ZA4744 Data file Version 5.0.0, doi:10.4232/1.11755

European Commission (2012): Eurobarometer 70.1 (Oct-Nov 2008). TNS OPINION & SOCIAL, Brussels [Producer]. GESIS Data Archive, Cologne. ZA4819 Data file Version 3.0.2, doi:10.4232/1.10989

European Commission (2013): Eurobarometer 71.1 (Jan-Feb 2009). TNS OPINION & SOCIAL, Brussels [Producer]. GESIS Data Archive, Cologne. ZA4971 Data file Version 4.0.0, doi:10.4232/1.11756

European Commission (2012): Eurobarometer 71.2 (May-Jun 2009). TNS OPINION & SOCIAL, Brussels [Producer]. GESIS Data Archive, Cologne. ZA4972 Data file Version 3.0.2, doi:10.4232/1.10990

European Commission (2012): Eurobarometer 71.3 (Jun-Jul 2009). TNS OPINION & SOCIAL, Brussels [Producer]. GESIS Data Archive, Cologne. ZA4973 Data file Version 3.0.0, doi:10.4232/1.11135

European Commission (2012): Eurobarometer 73.5 (Jun 2010). TNS OPINION & SOCIAL, Brussels [Producer]. GESIS Data Archive, Cologne. ZA5235 Data file Version 4.0.0, doi:10.4232/1.11432

European Commission (2013): Eurobarometer 74.2 (2010). TNS OPINION & SOCIAL, Brussels [Producer]. GESIS Data Archive, Cologne. ZA5449 Data file Version 2.2.0, doi:10.4232/1.11626

*Table A2. Empirical association between the measures of individual socioeconomic status (% of respondents).*

| \|  \| \| ***Professional /manager and business owner*** \| \|  \| \| --- \| --- \| --- \| --- \| --- \| \| *No* \| *Yes* \| *Total* \| \| ***Tertiary education*** \| *No* \| 62.67 \| 1.74 \| 64.41 \| \| *Yes* \| 32.94 \| **2.66** \| 35.59 \| \|  \| *Total* \| 95.60 \| 4.40 \| 100.00 \| | \|  \| \| ***Apartment ownership l l*** \| \|  \| \| --- \| --- \| --- \| --- \| --- \| \| *No* \| *Yes* \| *Total* \| \| ***Tertiary education*** \| *No* \| 30.90 \| 33.62 \| 64.52 \| \| *Yes* \| 21.09 \| **14.40** \| 35.48 \| \|  \| *Total* \| 51.98 \| 48.02 \| 100.00 \| |
| --- | --- | --- | --- | --- | --- | --- | --- | --- | --- | --- | --- | --- | --- | --- | --- | --- | --- | --- | --- | --- | --- | --- | --- | --- | --- | --- | --- | --- | --- | --- | --- | --- | --- | --- | --- | --- | --- | --- | --- | --- | --- | --- | --- | --- | --- |
| Pearson's correlation: 0.111*** | Pearson's correlation: -0.111*** |
| \|  \| \| ***No difficulty with bills l*** \| \|  \| \| --- \| --- \| --- \| --- \| --- \| \| *No* \| *Yes* \| *Total* \| \| ***Tertiary education*** \| *No* \| 31.23 \| 33.18 \| 64.41 \| \| *Yes* \| 11.59 \| **23.99** \| 35.59 \| \|  \| *Total* \| 42.83 \| 57.17 \| 100.00 \| | \|  \| \| ***Professional /manager and business owner l*** \| \|  \| \| --- \| --- \| --- \| --- \| --- \| \| *No* \| *Yes* \| *Total* \| \| ***Apartment ownership*** \| *No* \| 49.50 \| 2.48 \| 51.98 \| \| *Yes* \| 46.09 \| **1.92** \| 48.02 \| \|  \| *Total* \| 95.59 \| 4.41 \| 100.00 \| |
| Pearson's correlation: 0.154*** | Pearson's correlation: -0.019*** |
| \|  \| \| ***Professional /manager and business owner*** \| \|  \| \| --- \| --- \| --- \| --- \| --- \| \| *No* \| *Yes* \| *Total* \| \| ***No diffic. with bills*** \| *No* \| 41.57 \| 1.25 \| 42.83 \| \| *Yes* \| 53.92 \| **3.25** \| 57.17 \| \|  \| *Total* \| 95.49 \| 4.51 \| 100.00 \| | \|  \| \| ***No difficulty with bills l*** \| \|  \| \| --- \| --- \| --- \| --- \| --- \| \| *No* \| *Yes* \| *Total* \| \| ***Apartment ownership*** \| *No* \| 24.30 \| 28.81 \| 53.11 \| \| *Yes* \| 18.97 \| **27.92** \| 46.89 \| \|  \| *Total* \| 43.27 \| 56.73 \| 100.00 \| |
| Pearson's correlation: 0.066*** | Pearson's correlation: 0.054*** |

*Note: data in the table are reported in percentage. Absolute total values are not the same across couples of variables, since the questions about apartment ownership and the difficulty in paying the bills were not asked in all the surveys employed in the present analysis (see Table 2 in the main paper, comparing the total number of observations from column [c] to column [d]).* *** p<0.01

*Table A3. Source and description of the independent variables.*

| **Name** | **Description** | **Source** | **Year** |
| --- | --- | --- | --- |
| ***Individual characteristics (survey data)*** | | | |
| *Tertiary education* | Dummy equal to 1 if the respondent completed tertiary education | Eurobarometer | *2004-2010* |
| *Apartment ownership* | Dummy equal to 1 if the respondent owns the apartment she/he is living in | Eurobarometer | *2005-2010* |
| *No difficulty with bills* | Dummy equal to 1 if the respondent did never encountered difficulties in paying the bills in the last 12 months | Eurobarometer | *2009-2010* |
| *Make ends meet* | Dummy equal to 1 if the respondent did not have difficulties in making ends meet at the end of the month | Eurobarometer | *2008-2009* |
| *Occupation* | Occupation of the respondent: professional/manager and business owner, shop owner, employee, farmer/ fisherman, house person, student, retired, manual worker, unemployed (reference category = unemployed) | Eurobarometer | *2004-2010* |
| *Age* | Age of the respondent (number of years) | Eurobarometer | *2004-2010* |
| *Female* | Dummy equal to 1 if the respondent is a woman | Eurobarometer | *2004-2010* |
| *Children* | Number of children in the household | Eurobarometer | *2004-2010* |
| *Marital status* | Marital status of the respondent: single, married, separated/ divorced, widower (reference category = married) | Eurobarometer | *2004-2010* |
| ***Urban variables (NUTS3)*** | | | |
| *Per capita GDP* | Per capita real GDP in the NUTS3 region of residence | Eurostat | *2004-2010* |
| *City ranking^[[1]](#footnote-1)^* | NUTS3 regions are classified into six mutually exclusive categories: 1^st^ rank (more than 1 million inhabitants; 24 regions), 2^nd^ rank (500,000-1 million; 44 regions), 3^rd^ rank (250-500,000; 98 regions), 4^th^ rank (100-250,000; 304 regions), 5^th^ rank (50-100,000; 311 regions) and 6^th^ rank (less than 50,000; 514 regions) | Eurostat | *2004-2010* |
| *Distance from the closest 1^st^ or 2^nd^ rank city* | Distance from the closest 1^st^ or 2^nd^ rank city, measured in terms of travel time by car | Google Maps | *2004-2010* |
| ***Time controls*** | | | |
| *EB waves* | Set of dummy variables for the different EB survey studies employed in the analysis | Eurobarometer | *2004-2010* |

T*able A4. Life satisfaction: the interplay between urbanisation, education, occupation and income.*

|  | [a] | [b] | [c] | [d] | [e] |
| --- | --- | --- | --- | --- | --- |
| Level 1: individual characteristics |  |  |  |  |  |
| **Tertiary education** | 0.112*** | 0.108*** | 0.112*** | 0.110*** | 0.094*** |
|  | (0.003) | (0.003) | (0.003) | (0.004) | (0.006) |
| **Apartment ownership** |  |  |  | 0.100*** |  |
|  |  |  |  | (0.004) |  |
| **No difficulty with bills** |  |  |  |  | 0.333*** |
|  |  |  |  |  | (0.006) |
| **Professional /manager /business owner** | 0.358*** | 0.358*** | 0.355*** | 0.338*** | 0.407*** |
|  | (0.008) | (0.008) | (0.008) | (0.009) | (0.015) |
| Shop owner | 0.265*** | 0.265*** | 0.265*** | 0.250*** | 0.354*** |
|  | (0.009) | (0.009) | (0.009) | (0.010) | (0.017) |
| Employee | 0.282*** | 0.282*** | 0.282*** | 0.270*** | 0.363*** |
|  | (0.005) | (0.005) | (0.005) | (0.005) | (0.010) |
| Farmer/ fisherman | 0.154*** | 0.153*** | 0.154*** | 0.130*** | 0.244*** |
|  | (0.013) | (0.013) | (0.013) | (0.015) | (0.027) |
| Manual worker | 0.162*** | 0.162*** | 0.162*** | 0.147*** | 0.272*** |
|  | (0.005) | (0.005) | (0.005) | (0.006) | (0.011) |
| Houseperson | 0.111*** | 0.111*** | 0.111*** | 0.087*** | 0.217*** |
|  | (0.005) | (0.005) | (0.005) | (0.006) | (0.014) |
| Student | 0.273*** | 0.272*** | 0.273*** | 0.225*** | 0.412*** |
|  | (0.008) | (0.008) | (0.008) | (0.009) | (0.015) |
| Retired | 0.145*** | 0.145*** | 0.145*** | 0.127*** | 0.221*** |
|  | (0.005) | (0.005) | (0.005) | (0.006) | (0.011) |
| Age | -0.024*** | -0.024*** | -0.024*** | -0.026*** | -0.019*** |
|  | (0.001) | (0.001) | (0.001) | (0.001) | (0.001) |
| Age^2 | 0.000*** | 0.000*** | 0.000*** | 0.000*** | 0.000*** |
|  | (0.000) | (0.000) | (0.000) | (0.000) | (0.000) |
| Female | 0.014*** | 0.014*** | 0.014*** | 0.016*** | 0.022*** |
|  | (0.003) | (0.003) | (0.003) | (0.003) | (0.005) |
| Children | 0.002 | 0.002 | 0.002 | 0.005** | 0.019*** |
|  | (0.002) | (0.002) | (0.002) | (0.003) | (0.004) |
| Single | -0.118*** | -0.118*** | -0.118*** | -0.116*** | -0.073*** |
|  | (0.004) | (0.004) | (0.004) | (0.004) | (0.006) |
| Divorced | -0.255*** | -0.255*** | -0.255*** | -0.236*** | -0.180*** |
|  | (0.005) | (0.005) | (0.005) | (0.006) | (0.010) |
| Widower | -0.208*** | -0.208*** | -0.208*** | -0.200*** | -0.152*** |
|  | (0.005) | (0.005) | (0.005) | (0.006) | (0.010) |
| Level 2: regional characteristics |  |  |  |  |  |
| GDP region | 0.022*** | 0.022*** | 0.022*** | 0.018*** | 0.003 |
|  | (0.006) | (0.006) | (0.006) | (0.007) | (0.009) |
| 5^th^ rank cities | -0.026** | -0.026** | -0.026** | -0.019 | 0.003 |
|  | (0.012) | (0.012) | (0.012) | (0.013) | (0.017) |
| 4^th^ rank cities | -0.010 | -0.010 | -0.010 | -0.005 | 0.011 |
|  | (0.013) | (0.013) | (0.013) | (0.014) | (0.018) |
| 3^rd^ rank cities | -0.009 | -0.009 | -0.009 | 0.005 | 0.016 |
|  | (0.019) | (0.019) | (0.019) | (0.019) | (0.024) |
| 2^nd^ rank cities | -0.043* | -0.042* | -0.043* | -0.027 | -0.012 |
|  | (0.024) | (0.024) | (0.024) | (0.025) | (0.030) |
| **1^st^ rank cities** | **-0.068**** | **-0.085***** | **-0.070**** | **-0.055*** | **-0.054** |
|  | **(0.029)** | **(0.030)** | **(0.029)** | **(0.031)** | **(0.039)** |
| **1^st^ rank * tertiary education** |  | **0.046***** |  |  |  |
|  |  | **(0.011)** |  |  |  |
| **1^st^ rank * Professional /manager /business owner** |  |  | **0.040*** |  |  |
|  |  |  | **(0.022)** |  |  |
| **1^st^ rank * apartment owner** |  |  |  | **0.027**** |  |
|  |  |  |  | **(0.013)** |  |
| **1^st^ rank * no difficulty with bills** |  |  |  |  | **0.070***** |
|  |  |  |  |  | **(0.019)** |
| EB wave dummies | Yes | Yes | Yes | Yes | Yes |
| Constant | 3.301*** | 3.302*** | 3.301*** | 3.338*** | 2.912*** |
|  | (0.090) | (0.090) | (0.090) | (0.096) | (0.088) |
| Random effects |  |  |  |  |  |
| Level 1 (individual) variance | 0.442 | 0.442 | 0.442 | 0.436 | 0.418 |
|  | 0.001 | 0.001 | 0.001 | 0.001 | 0.002 |
| Level 2 (region) variance | 0.011 | 0.011 | 0.011 | 0.011 | 0.014 |
|  | 0.001 | 0.001 | 0.001 | 0.001 | 0.001 |
| Level 3 (country) variance | 0.141 | 0.141 | 0.141 | 0.16 | 0.123 |
|  | 0.047 | 0.047 | 0.047 | 0.054 | 0.041 |
| ICC – level 2 (region) | 0.019 | 0.019 | 0.019 | 0.018 | 0.025 |
| ICC – level 3 (country) | 0.237 | 0.237 | 0.237 | 0.264 | 0.222 |
| Observations | 252,271 | 252,271 | 252,271 | 186,066 | 68,315 |

Reference categories: unemployed (occupation), married (marital status), 6^th^ rank cities (city rank).

Standard errors in parentheses. *** p<0.01, ** p<0.05, * p<0.1.

*Table A5. Distance from 1^st^ rank cities and life satisfaction: the role of education, occupation and income.*

|  | [a] | [a1] | [a2] | [a3] | [a4] | [b1] | [c1] | [d1] | [e1] |
| --- | --- | --- | --- | --- | --- | --- | --- | --- | --- |
|  | Distance band from the closest 1^st^ rank NUTS3 | | | | | | | | |
|  | Whole sample | < 0.5 hours | 0.5 - 1.5 hours | 1.5 - 3 hours | > 3 hours | Whole sample | Whole sample | Whole sample | Whole sample |
| Level 1: individual characteristics |  |  |  |  |  |  |  |  |  |
| Tertiary education | 0.109*** | 0.174*** | 0.117*** | 0.139*** | 0.101*** | 0.122*** | 0.109*** | 0.109*** | 0.094*** |
|  | (0.003) | (0.038) | (0.009) | (0.008) | (0.004) | (0.005) | (0.003) | (0.004) | (0.007) |
| Apartment ownership |  |  |  |  |  |  |  | 0.134*** |  |
|  |  |  |  |  |  |  |  | (0.006) |  |
| No difficulty with bills |  |  |  |  |  |  |  |  | 0.353*** |
|  |  |  |  |  |  |  |  |  | (0.009) |
| Professional /manager and business owner | 0.357*** | 0.364*** | 0.336*** | 0.382*** | 0.353*** | 0.357*** | 0.359*** | 0.336*** | 0.416*** |
|  | (0.008) | (0.078) | (0.021) | (0.017) | (0.010) | (0.008) | (0.011) | (0.009) | (0.016) |
| Shop owner | 0.267*** | 0.229* | 0.261*** | 0.318*** | 0.254*** | 0.267*** | 0.267*** | 0.251*** | 0.365*** |
|  | (0.009) | (0.130) | (0.023) | (0.024) | (0.011) | (0.009) | (0.009) | (0.011) | (0.018) |
| Employee | 0.284*** | 0.279*** | 0.285*** | 0.303*** | 0.279*** | 0.284*** | 0.284*** | 0.273*** | 0.367*** |
|  | (0.005) | (0.057) | (0.012) | (0.011) | (0.006) | (0.005) | (0.005) | (0.005) | (0.011) |
| Farmer/ fisherman | 0.154*** | -0.099 | 0.167*** | 0.204*** | 0.141*** | 0.154*** | 0.154*** | 0.131*** | 0.245*** |
|  | (0.013) | (0.623) | (0.046) | (0.034) | (0.014) | (0.013) | (0.013) | (0.015) | (0.027) |
| Manual worker | 0.168*** | 0.116 | 0.198*** | 0.170*** | 0.162*** | 0.168*** | 0.168*** | 0.152*** | 0.278*** |
|  | (0.006) | (0.079) | (0.014) | (0.013) | (0.007) | (0.006) | (0.006) | (0.006) | (0.012) |
| Houseperson | 0.109*** | 0.126** | 0.098*** | 0.153*** | 0.097*** | 0.109*** | 0.109*** | 0.086*** | 0.216*** |
|  | (0.005) | (0.063) | (0.014) | (0.012) | (0.007) | (0.005) | (0.005) | (0.006) | (0.014) |
| Student | 0.272*** | 0.135 | 0.302*** | 0.285*** | 0.261*** | 0.272*** | 0.272*** | 0.227*** | 0.412*** |
|  | (0.008) | (0.091) | (0.022) | (0.020) | (0.009) | (0.008) | (0.008) | (0.009) | (0.015) |
| Retired | 0.149*** | 0.265*** | 0.147*** | 0.155*** | 0.144*** | 0.149*** | 0.149*** | 0.130*** | 0.227*** |
|  | (0.006) | (0.066) | (0.014) | (0.013) | (0.007) | (0.006) | (0.006) | (0.006) | (0.012) |
| Age | -0.024*** | -0.036*** | -0.022*** | -0.025*** | -0.024*** | -0.024*** | -0.024*** | -0.026*** | -0.019*** |
|  | (0.001) | (0.006) | (0.001) | (0.001) | (0.001) | (0.001) | (0.001) | (0.001) | (0.001) |
| Age^2 | 0.000*** | 0.000*** | 0.000*** | 0.000*** | 0.000*** | 0.000*** | 0.000*** | 0.000*** | 0.000*** |
|  | (0.000) | (0.000) | (0.000) | (0.000) | (0.000) | (0.000) | (0.000) | (0.000) | (0.000) |
| Female | 0.015*** | 0.065* | 0.020*** | 0.013* | 0.015*** | 0.015*** | 0.015*** | 0.017*** | 0.023*** |
|  | (0.003) | (0.034) | (0.008) | (0.007) | (0.004) | (0.003) | (0.003) | (0.003) | (0.005) |
| Children | 0.003 | -0.028 | 0.005 | -0.002 | 0.004 | 0.003 | 0.003 | 0.005** | 0.018*** |
|  | (0.002) | (0.025) | (0.006) | (0.005) | (0.003) | (0.002) | (0.002) | (0.003) | (0.004) |
| Single | -0.118*** | -0.195*** | -0.119*** | -0.127*** | -0.115*** | -0.118*** | -0.118*** | -0.116*** | -0.073*** |
|  | (0.004) | (0.045) | (0.010) | (0.009) | (0.005) | (0.004) | (0.004) | (0.005) | (0.007) |
| Divorced | -0.256*** | -0.321*** | -0.313*** | -0.287*** | -0.229*** | -0.256*** | -0.256*** | -0.238*** | -0.178*** |
|  | (0.005) | (0.062) | (0.013) | (0.012) | (0.007) | (0.005) | (0.005) | (0.006) | (0.011) |
| Widower | -0.211*** | -0.308*** | -0.262*** | -0.224*** | -0.194*** | -0.210*** | -0.211*** | -0.204*** | -0.151*** |
|  | (0.005) | (0.068) | (0.014) | (0.013) | (0.007) | (0.005) | (0.005) | (0.006) | (0.010) |
| Level 2: regional characteristics |  |  |  |  |  |  |  |  |  |
| GDP region | 0.023*** | 0.036* | 0.044*** | 0.027** | 0.014 | 0.022*** | 0.023*** | 0.017** | 0.006 |
|  | (0.007) | (0.020) | (0.015) | (0.012) | (0.011) | (0.007) | (0.007) | (0.007) | (0.010) |
| 5^th^ rank cities | -0.027** | -0.006 | -0.040 | -0.051* | -0.006 | -0.028** | -0.027** | -0.019 | 0.001 |
|  | (0.012) | (0.085) | (0.027) | (0.026) | (0.016) | (0.012) | (0.012) | (0.013) | (0.017) |
| 4^th^ rank cities | -0.011 | 0.043 | -0.070** | -0.020 | 0.013 | -0.011 | -0.011 | -0.003 | 0.009 |
|  | (0.013) | (0.170) | (0.029) | (0.028) | (0.018) | (0.013) | (0.013) | (0.014) | (0.018) |
| 3^rd^ rank cities | -0.009 |  | -0.095** | -0.059 | 0.021 | -0.010 | -0.009 | 0.008 | 0.013 |
|  | (0.019) |  | (0.043) | (0.039) | (0.024) | (0.019) | (0.019) | (0.019) | (0.024) |
| 2^nd^ rank cities | -0.045* |  | -0.172** | -0.089* | -0.012 | -0.045* | -0.045* | -0.025 | -0.019 |
|  | (0.024) |  | (0.072) | (0.047) | (0.030) | (0.024) | (0.024) | (0.025) | (0.031) |
| Distance to closest 1^st^ rank | -0.005*** | -0.597 | -0.078* | -0.071** | -0.005*** | -0.004*** | -0.005*** | -0.001 | -0.002 |
|  | (0.002) | (1.290) | (0.040) | (0.033) | (0.002) | (0.002) | (0.002) | (0.002) | (0.002) |
| Distance 1^st^ rank * tertiary education |  |  |  |  |  | -0.003*** |  |  |  |
|  |  |  |  |  |  | (0.001) |  |  |  |
| Distance 1^st^ rank * Professional /manager /business owner |  |  |  |  |  |  | -0.000 |  |  |
|  |  |  |  |  |  |  | (0.002) |  |  |
| Distance 1^st^ rank * apartment owner |  |  |  |  |  |  |  | -0.007*** |  |
|  |  |  |  |  |  |  |  | (0.001) |  |
| Distance 1^st^ rank * no difficulty with bills |  |  |  |  |  |  |  |  | -0.005*** |
|  |  |  |  |  |  |  |  |  | (0.001) |
| EB wave dummies | Yes | Yes | Yes | Yes | Yes | Yes | Yes | Yes | Yes |
| Constant | 3.319*** | 3.842*** | 3.237*** | 3.343*** | 3.323*** | 3.315*** | 3.319*** | 3.335*** | 2.933*** |
|  | (0.091) | (0.563) | (0.128) | (0.126) | (0.097) | (0.091) | (0.091) | (0.098) | (0.088) |
| Random effects |  |  |  |  |  |  |  |  |  |
| Level 1 (individual) variance | 0.440 | 0.376 | 0.450 | 0.440 | 0.436 | 0.440 | 0.440 | 0.433 | 0.417 |
|  | 0.001 | 0.014 | 0.003 | 0.003 | 0.002 | 0.001 | 0.001 | 0.001 | 0.002 |
| Level 2 (region) variance | 0.011 | 0.005 | 0.012 | 0.011 | 0.009 | 0.011 | 0.011 | 0.011 | 0.014 |
|  | 0.001 | 0.006 | 0.002 | 0.002 | 0.001 | 0.001 | 0.001 | 0.001 | 0.001 |
| Level 3 (country) variance | 0.143 | 0.030 | 0.133 | 0.111 | 0.151 | 0.143 | 0.143 | 0.163 | 0.122 |
|  | 0.048 | 0.028 | 0.059 | 0.049 | 0.052 | 0.048 | 0.048 | 0.055 | 0.041 |
| ICC – level 2 (region) | 0.019 | 0.011 | 0.021 | 0.019 | 0.015 | 0.019 | 0.019 | 0.018 | 0.025 |
| ICC – level 3 (country) | 0.241 | 0.072 | 0.224 | 0.197 | 0.253 | 0.241 | 0.241 | 0.269 | 0.221 |
| Observations | 233,966 | 1,524 | 35,713 | 43,828 | 152,901 | 233,966 | 233,966 | 172,159 | 62,857 |

Reference categories: unemployed (occupation), married (marital status), 6^th^ rank cities (city rank).

Standard errors in parentheses. *** p<0.01, ** p<0.05, * p<0.1.

*Table A6. Minimum distance from the closest first-rank city and life satisfaction across cities of different size.*

|  | [b] | [c] | [d] |
| --- | --- | --- | --- |
| Level 1: individual characteristics | Yes | Yes | Yes |
| Level 2: regional characteristics |  |  |  |
| GDP region | 0.032*** | 0.032*** | 0.032*** |
|  | (0.007) | (0.007) | (0.007) |
| 5^th^ rank cities | -0.030** | -0.030** | -0.042** |
|  | (0.012) | (0.012) | (0.018) |
| 4^th^ rank cities | -0.015 | -0.025 | -0.014 |
|  | (0.013) | (0.016) | (0.013) |
| 3^rd^ rank cities | -0.010 | -0.011 | -0.012 |
|  | (0.022) | (0.019) | (0.019) |
| Distance to closest 1^st^ or 2^nd^ rank | -0.005** | -0.007*** | -0.005*** |
|  | (0.002) | (0.003) | (0.002) |
| Distance to 1^st^ or 2^nd^ rank * 3^rd^ rank cities | -0.001 |  |  |
|  | (0.004) |  |  |
| Distance to 1^st^ or 2^nd^ rank * 4^th^ rank cities |  | 0.004 |  |
|  |  | (0.003) |  |
| Distance to 1^st^ or 2^nd^ rank * 5^th^ rank cities |  |  | 0.006 |
|  |  |  | (0.006) |
| EB wave dummies | Yes | Yes | Yes |
| Constant | 3.302*** | 3.307*** | 3.302*** |
|  | (0.089) | (0.090) | (0.089) |
| Observations | 204,316 | 204,316 | 204,316 |

1. More specifically, the 1^st^ rank EU NUTS3 regions are the following ones: AT130 (Wien), BE100 (Arr. de Bruxelles), BG411 (Sofia), CZ010 (Praha), ES300 (Madrid), ES511 (Barcelona), FR101 (Paris), FR301 (Nord), FR716 (Rhône), FR825 (Var), HU101 (Budapest), ITC4C (Milano), ITI43 (Roma), PL127 (Miasto Warszawa), PL22A (Katowicki), RO321 (Bucuresti), UKD32 (Greater Manchester North), UKD72 (Liverpool), UKG31 (Birmingham), UKI3 and UKI4 (Inner London), DE212 (München), DE300 (Berlin), DE600 (Hamburg), DEA23 (Köln). As reported in the main paper (footnote 5), EB surveys do not provide data at the NUTS3 level for UK, Finland, Poland, Czech Republic, Estonia, Lithuania and Slovakia. Therefore, these countries are excluded from the analysis. However, we considered the major cities of these countries when calculating the travel time distance for individuals living in other regions. For instance, a respondent living in Eastern Germany might be closer to a Polish 1^st^ rank city rather than to a German one. Based on the same reasoning, we considered in our analysis also the distance to non-EU large cities of either first, second or third-rank. First-rank, non-EU cities are Belgrad (RS), Zurich (CH), Instanbul (TR), St. Petersburg (RU), Kiev (UA) and Minsk (BY). [↑](#footnote-ref-1)
